# The association between sarcopenia and clinical outcomes among Chinese patients with triple-negative breast cancer: a retrospective study

**DOI:** 10.3389/fonc.2025.1402300

**Published:** 2025-02-06

**Authors:** Huayan Gu, Teng Zhu, JiaLing Ding, Zhi Yang, Yiqiao Lu, Guilong Guo

**Affiliations:** ^1^ Department of Breast Surgery, First Affiliated Hospital of Wenzhou Medical University, Wenzhou, Zhejiang, China; ^2^ Department of Gastrointestinal Surgery, First Affiliated Hospital of Wenzhou Medical University, Wenzhou, Zhejiang, China

**Keywords:** sarcopenia, triple-negative breast cancer, TNBC, association, clinical outcomes

## Abstract

**Purpose:**

This study efforts to explore the association between sarcopenia, an age-related decline in muscle mass and physical function, and clinical outcomes in women with triple-negative breast cancer (TNBC).

**Methods and materials:**

This retrospective study included women diagnosed with TNBC who received breast surgery from December 2012 to December 2018. Skeletal muscle mass index (SMI) is utilized to evaluate sarcopenia, which was quantified by the cross-sectional area of muscle at the twelfth thoracic vertebra (T12). Disease-free survival (DFS) and overall survival (OS) are the endpoints. The association of sarcopenia with DFS and OS was statistically analyzed.

**Results:**

The study included 130 women in all, with a median age of 55 years (median follow-up, 53 months). Among them, 78 (58.5%) women had sarcopenia (SMI <28.01). 38 patients (29.2%) died and 49 patients (37.7%) experienced a recurrence of breast cancer throughout the follow-up period. Sarcopenia was demonstrated to be a significant predictive factor for both OS (HR,2.885; 95% CI, 1.349–6.169; p = 0.006) and DFS (HR,3.121; 95% CI, 1.578–6.175; p = 0.001) in the multivariate Cox proportional hazard model. There was no significant correlation seen between body mass index and either DFS (p = 0.156) or OS (P = 0.264). Logistic regression model further revealed that sarcopenia was a prognostic factor that was independently associated with both DFS (p = 0.001) and OS (p = 0.006).

**Conclusions:**

Among women with TNBC, sarcopenia is associated with worse clinical outcomes. These patients with high risk might be candidates for individual programmed exercise and diet interventions to optimize survival outcomes.

## Introduction

Breast cancer (BC) is the most frequent type cancer in women, in 2022, there were around 2.8 million new cases worldwide, accounting for about 25% of all female cancers, while it surpassed lung cancer cases of 2.3 million, making breast cancer the number one cancer worldwide (1). The prognosis for breast cancer has significantly improved due to advancements in diagnosis and treatment. For all stages combined, the five-year survival rate is close to 90%. About 15 to 20 percent of breast tumors are triple-negative breast cancers (TNBC), which are defined by the absence of the progesterone receptor (PR), estrogen receptor (ER) and human epidermal growth factor receptor 2(HER2). Compared with other subtypes, it has the worst prognosis. After diagnosis, almost 50% of patients experience relapses during the first 3 to 5 years (2). At the same time, TNBC is highly aggressive and has the highest probability of distant metastasis (~46%) (3, 4). Additionally, there was only a 13.3-month median survival following distant metastases (4).

Consequently, in order to customize treatment for TNBC patients, it is vital to make more accurate prognostic predictions. Numerous prognostic factors have been found and are classified as follows: tumor characteristics including size, stage, grade, and involvement of lymph nodes (5); host characteristics such as sarcopenia, age, race, and financial condition (6) and treatment characteristics such as type of chemotherapy (7). Among host characteristics, sarcopenia is becoming more widely recognized as a factor that predicts the patients’ death from breast cancer (8).

Sarcopenia, which is associated with reduced skeletal muscle mass, strength, and function in cancer patients, is significantly linked to physical impairment, quality of life and mortality (9). In 2018, the European Working Group on Sarcopenia in Older People (EWGSOP) updated the original definition. They focused on low muscle strength as a key characteristic of sarcopenia, used detection of low muscle quantity and quality to confirm the sarcopenia diagnosis, and identified poor physical performance as indicative of severe sarcopenia. A wide range of tests and tools are now available to characterize sarcopenia in practice and research, including measurement of muscle strength using grip strength and chair-stand tests, and measurement of muscle mass using Dual-energy X-ray absorptiometry (DXA), bioelectrical impedance analysis (BIA), Magnetic resonance imaging (MRI), computed tomography (CT) and so on. In addition, MRI and CT are considered the gold standards for non-invasive muscle quantity/mass assessment, which are more readily available in the hospital setting than other methods (10). It is known that CT-based regional analysis of muscle and fat tissue in the area of the third lumbar vertebra level (L3) is tied to whole-body fat and muscle mass. But for breast cancer, thoracic CT slices have been used for body composition analysis as their CT imaging may not include slices from the lumbar region (11). Some studies have explored the findings that skeletal muscle measurements at the T12 level could be used as an alternative method to objectively define sarcopenia in patients without abdominal CT imaging (12–14).

Since the measurements of body composition in sarcopenia can act as indicators of general health, they may consequently be crucial in the prediction of disease processes. Sarcopenia breast cancer patients had a lower overall survival than non-sarcopenic patients, according to recent studies (HR = 2.86; 95% CI, 1.67–4.89) (15). To the best of our knowledge, nevertheless, there is no evidence to assess sarcopenia’s prognostic significance in TNBC.

The objective of this retrospective study was to evaluate the impact of sarcopenia in Chinese patients with triple-negative breast cancer, in order to provide timely intervention for better clinical outcomes.

## Materials and methods

### Participants

This retrospective study was performed at the First Affiliated Hospital of Wenzhou Medical University on patients treated from December 2012 to December 2018. They were chosen from the institution’s computerized database based on the specified inclusion criteria: (1) at diagnosis, female patients were over 18 years old, (2) they were diagnosed with TNBC through biopsy pathology and immunohistochemistry, (3) patients had comprehensive clinical and follow-up information, and (4) CT scan imaging was conducted in the hospital prior to the operation and stored in the institution’s radiology database. The exclusion criteria contained the following: (1) patients with metastatic disease, (2) inadequate clinical data, chest CT images, and lack of follow-up.

Data on survival were acquired via phone conversations and/or outpatient medical records. From the medical and imaging records of each individual patient, information regarding recurrence and metastasis was acquired. The period from the date of operation to the date of metastasis, recurrence, and/or last follow-up was used to compute DFS. OS was defined as the interval between the date of operation and the patient’s death or the last follow-up.

The study was approved by the Institutional Ethics Review Board of the First Affiliated Hospital of Wenzhou Medical University. Informed consent was also signed by patients for the use of clinical data and biological sample data.

### Data collection

The following initial patient details were gathered: medical history, time of operation, menstruation status, tumor size, metastasis to lymph nodes, histological type, proliferation index (Ki67) expression, and hematological tests. Specialized breast cancer nurses measured patients’ body composition primarily when they were in the hospital. By dividing weight (kg) by height squared (m^2^), one can obtain BMI (kg/m^2^). The prognostic nutritional index (PNI), which is a comprehensive index to evaluate the nutritional status of patients (16), was calculated as follows: serum albumin concentration (g/dL) +5× peripheral lymphocyte count (number/mm^2^).

### CT-based skeletal muscle mass measures

Eligible chest CT images were assessed. Analyses were conducted with the guidance of a faculty Radiologist. The transverse section at the level of T12 was extracted for analysis.

**Figure 1:**
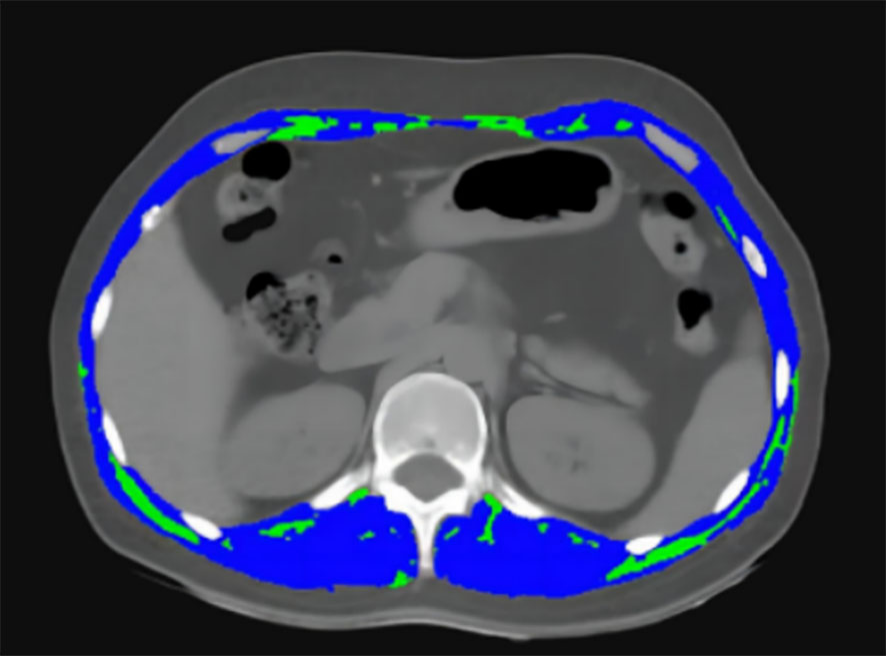
The image depicts the transverse section at the level of T12 on chest CT images. Sarcopenia was measured using the combined area of multiple skeletal muscles at T12, including the rectus abdominis, external oblique, internal oblique, latissimus dorsi, intercostal, and erector spinae muscles (blue). The intramuscular adipose tissue (IMAT) was showed as green area.

At the twelfth thoracic vertebra (T12), the cross-sectional area of muscle and intermuscular adipose tissue was measured in centimeters squared using SliceOmatic Software version 5.0 (v.4.3 Tomovision, Montreal, Canada), using *a priori* information about the T12 muscle shape to provide a highly accurate estimation of muscle and discriminating components by tissue-specific Hounsfield units (HU) ranges (17) (**Figure 1**). Previous research indicates that the HU thresholds for skeletal muscles were set between -29 and +150, and for fat tissues, between -150 and -50 (18, 19). From this software, the skeletal muscle area (SMA) was calculated.

Muscle area at T12 in cm^2^ divided by height in meters squared yields the skeletal muscle index (SMI). The average radiation attenuation of tissue in HU is used to calculate skeletal muscle radiodensity (SMD), a measure of muscle quality. Intramuscular adipose tissue (IMAT) was quantified separately (**Figure 1**). In order to express the measured total cross-section (cm^2^) in values of cm^2/^m^2^, it was normalized for meters squared (m^2^).

Due to the lack of agreement, we used the previously reported definition of cut-offs: sarcopenia is defined as T12 skeletal muscle index < 28.01 cm^2^/m^2^ (13).

### Statistical analysis

The follow-up period started on the day of the surgery and lasted until January 1, 2023, the last contact, or death. Qualitative outcomes were presented as percentages, while quantitative results were presented as median ± SD. For continuous data, the t test was used to assess group differences; for categorical variables, chi-square or Fisher’s exact test was employed. To obtain survival curves, the Kapla-Meier technique was employed. Cox proportional hazards regression was used to assess factors (p ≤ 0.1) from the univariate analysis in a multivariate model. Meanwhile, hazard ratios (HRs) and 95% confidence intervals (CIs) were computed. The following were the primary factors of interest: age, menopausal status, lump size, number of positive lymph nodes, Ki67 status, SMI, IMAT and PNI. Moreover, the logistic regression model was used to identify the independent affecting elements.

With SPSS version 26.0 software (SPSS Inc., Chicago, IL, USA), all statistical tests were performed. For statistical significance, a two-tailed P value of less than 0.05 was set.

## Results

### Characteristics of the patients

This study comprised 130 patients who met the inclusion and exclusion criteria between December 2012 and December 2018. A median follow-up time of 53 months (range, 1 to 93 months) was observed for the patients till January 1, 2023. 12 cases were lost to follow-up, yielding a 90.7% follow-up rate. The absence of an outpatient follow-up record and failure to contact were the primary causes of loss to follow-up. 38 patients (29.2%) passed away and 49 patients (37.7%) experienced a recurrence of breast cancer during the follow-up period.

The median age for all patients was 55 years (range, 28–88) and the average SMI of all patients was 26.7 cm^2^/m^2^. 76 patients (58.5%) had sarcopenia (SMI <28.01). Between patients with and without sarcopenia, there were notable variations in IMAT and BMI. Patients with sarcopenia had significantly decreased BMI (p < 0.001) and IMAT (p < 0.05) in comparison to those without the condition. **Table 1** contains a collection of more specific details.

**Table 1:** Baseline characteristics of TNBC patients stratified by SMI.

Variables	Sarcopenia (SMI<28.01)	Non-sarcopenia (SMI≥28.01)	p value
N	76	54	
Age (years)	55.7 ± 10.2	55.1 ± 10.0	0.733
Menopausal status			0.584
Pre	26 (34.2)	21 (38.9)	
Post	50 (65.8)	33 (61.1)	
BMI (kg/m^2^)	22.6 ± 2.9	26.9 ± 3.5	<0.001
Tumor size (cm)	2.5 ± 1.4	2.6 ± 1.4	0.600
Number of positive lymph nodes	2.9 ± 5.7	2.4 ± 5.5	0.627
Ki-67 (%)	58.9 ± 25.1	60.9 ± 24.8	0.653
SMA (cm^2^)	59.4 ± 8.2	75.0 ± 8.2	<0.001
SMI (cm^2^/m^2^)	23.8 ± 2.9	30.8 ± 2.7	<0.001
SMD (HD)	31.4 ± 7.9	31.9 ± 6.9	0.753
IMAT	5.4 ± 4.5	6.9 ± 3.7	0.035
PNI	50.9 ± 4.8	51.1 ± 4.6	0.788
DFS (months)	38.0 ± 22.4	51.1 ± 20.2	0.001
OS (months)	45.7 ± 20.6	54.1 ± 16.5	0.011

BMI, body mass index; SMA, Skeletal Muscle Area; SMI, Skeletal Muscle Index; SMD, Skeletal Muscle Radiodensity; IMAT, Intramuscular Adipose Tissue; PNI, Prognostic Nutritional Index; DFS, Disease-free Survival; OS, Overall Survival.

### Relationship between sarcopenia and DFS

**Figure 2:**
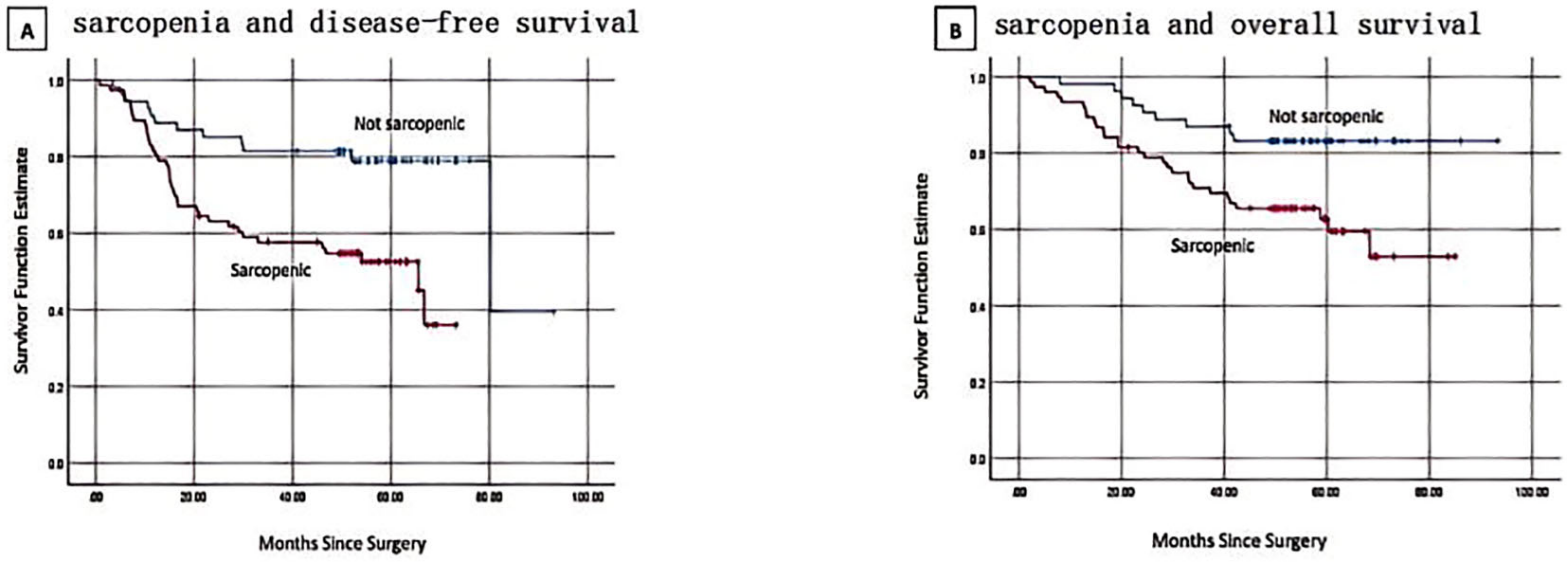
Kaplan-Meier curves show progression and survival rates for patients with sarcopenia. **(A)** sarcopenia and disease-free survival. log-rank p = 0.001 **(B)** sarcopenia and overall survival. log-rank p = 0.008.

There were 49 DFS occurrences and 38 reported deaths overall. Of the 49 DFS occurrences, 37 patients experienced distant metastases, while 12 patients experienced locoregional recurrence. Sarcopenia patients (SMI <28.01 cm^2^/m^2^) had a considerably shorter DFS than non-sarcopenic patients, according to Kaplan-Meier curves (log-rank p=0.001) (**Figure 2A**). In the univariate analysis, sarcopenia, SMD, age, menopausal state, lump size, numbers of positive lymph nodes, Ki67 status and PNI were discovered as significant factors. There was no discernible correlation between BMI and DFS (p = 0.224). The aforementioned variables were integrated into a multivariate Cox proportional hazard model. PNI was not substantially correlated with DFS (p = 0.361), and sarcopenia was strongly linked to worse DFS (HR, 3.121; 95% CI, 1.578-6.175; p = 0.001) (**Table 2**). Logistic regression model was founded, which revealed that sarcopenia remained independent prognostic factors associated with DFS (HR, 5.472; 95% CI, 2.019-14.831; p = 0.001) (**Table 3**).

**Table 2:** Univariate and multivariate analysis of factors associated with DFS and OS using Cox proportional hazards regression.

Factors	DFS				OS			
	Univariate analysis		Multivariate analysis		Univariate analysis		Multivariate analysis	
	HR (95% CI)	p value	HR (95% CI)	p value	HR (95% CI)	p value	HR (95% CI)	p value
Age (years)	1.036 (1.006-1.066)	0.017	1.022 (0.975-1.071)	0.366	1.044 (1.010-1.080)	0.011	1.050 (0.994-1.110)	0.081
Menopausal status		0.030		0.458		0.040		0.685
Pre	1 [Reference]		1 [Reference]		1 [Reference]		1 [Reference]	
Post	2.058 (1.072-3.951)		1.356 (0.607-3.027)		2.263 (1.037-4.938)		1.211 (0.481-3.051)	
BMI (kg/m^2^)	0.943 (0.869-1.023)	0.156	–	–	0.948 (0.863-1.041)	0.264	–	–
Tumor size (cm)	1.302 (1.113-1.523)	0.001	1.352 (1.119-1.634)	0.002	1.396 (1.184-1.647)	<0.001	1.578 (1.279-1.947)	<0.001
Number of positive lymph nodes	1.064 (1.030-1.099)	<0.001	1.055 (1.018-1.093)	0.003	1.071 (1.035-1.110)	<0.001	1.065 (1.023-1.108)	0.002
Ki-67 (%)	0.990 (0.979-1.001)	0.067	0.995 (0.982-1.008)	0.457	0.990 (0.978-1.002)	0.102	0.998 (0.983-1.012)	0.748
SMI (cm^2^/m^2^)		0.002		0.001		0.011		0.006
<28.01 (Sarcopenia)	2.968 (1.509-5.837)		3.121 (1.578-6.175)		2.652 (1.255-5.604)		2.885 (1.349-6.169)	
≥28.01 (Nonsarcopenia)	1 [Reference]		1 [Reference]		1 [Reference]		1 [Reference]	
SMD (HD)	0.968 (0.932-1.005)	0.089	0.988 (0.948-1.031)	0.585	0.964 (0.924-1.005)	0.087	0.989 (0.944-1.036)	0.634
IMAT	1.038 (0.978-1.101)	0.219	–	–	1.036 (0.968-1.108)	0.305	–	–
PNI	0.922 (0.869-0.978)	0.007	0.959 (0.895-1.028)	0.239	0.943 (0.884-1.006)	0.076	1.000 (0.926-1.080)	0.996

BMI, body mass index; SMI, Skeletal Muscle Index; SMD, Skeletal Muscle Radiodensity; IMAT, Intramuscular Adipose Tissue; PNI, Prognostic Nutritional Inde; DFS, Disease-free Survival; OS, Overall Survival; HR, Hazard Ratio.

**Table 3:** Analysis of factors associated with DFS and OS using Logistic regression.

Factors	DFS		OS	
	HR (95% CI)	p value	HR (95% CI)	p value
Age (years)	1.017 (0.955-1.083)	0.601	1.037 (0.971-1.107)	0.280
Menopausal status		0.635		0.555
Pre	1 [Reference]		1 [Reference]	
Post	1.323 (0.416-4.207)		1.467 (0.411-5.245)	
Tumor size (cm)	1.514 (1.074-2.133)	0.018	1.745 (1.233-2.469)	0.002
Number of positive lymph nodes	1.134 (1.022-1.259)	0.018	1.102 (1.007-1.205)	0.034
Ki-67 (%)	0.989 (0.969-1.009)	0.268	0.994 (0.975-1.014)	0.577
SMI (cm^2^/m^2^)		0.001		0.006
<28.01 (Sarcopenia)	5.472 (2.019-14.831)		4.315 (1.526-12.203)	
≥28.01 (Nonsarcopenia)	1 [Reference]		1 [Reference]	
SMD (HD)	1.043 (0.959-1.134)	0.328	1.021 (0.939-1.111)	0.629
IMAT	1.135 (0.981-1.313)	0.090	1.075 (0.937-1.233)	0.300
PNI	0.903 (0.816-1.000)	0.05	0.983 (0.890-1.087)	0.743

SMI, Skeletal Muscle Index; SMD, Skeletal Muscle Radiodensity; IMAT, Intramuscular Adipose Tissue; PNI, Prognostic Nutritional Inde; DFS, Disease-free Survival; OS, Overall Survival; HR, Hazard Ratio.

### Relationship between sarcopenia and OS

By the time the follow-up period ended, 38 deaths had been reported. Sarcopenic patients exhibited a significantly lower overall survival than non-sarcopenic patients, according to the Kaplan-Meier analysis (log-rank p=0.008, **Figure 2B**). Sarcopenia, SMD, age, menopausal status, lump size, number of positive lymph nodes, Ki67 status, and PNI were found to be predictive variables for OS in TNBC patients by the univariate Cox proportional hazard model analysis. Sarcopenic patients were found to have a significantly greater risk of death (HR, 2.885; 95% CI, 1.349–6.169; p = 0.006) in the multivariate analysis when compared to non-sarcopenic patients (**Table 2**). According to a logistic regression model, sarcopenia was found to be an independent prognostic factor linked with OS (HR, 4.315; 95% CI, 1.526-12.203; p = 0.006) (**Table 3**).

## Discussion

As far as we are aware, no other research has explicitly examined the relationship between sarcopenia and clinical outcomes in Chinese women with TNBC. Among patients diagnosed with TNBC, we discovered that sarcopenia was linked to a higher risk of recurrence as well as mortality.

There have been many previous studies on the prognosis and survival of breast cancer with sarcopenia (8, 20–23), but there have been few studies on the molecular subtypes of breast cancer. TNBC is more aggressive and malignant than other subtypes, has shorter recurrence-free survival and lacks effective therapeutic targets. Consequently, we had a strong desire to investigate the predictive significance of sarcopenia in patients with TNBC, since this can aid in the identification of high-risk populations and direct interventions to optimize survival outcomes.

Sarcopenia is usually diagnosed by the SMI at the L3 level from a CT scan, and there is no uniform threshold for sarcopenia. For individuals with breast cancer, abdomen CT is not an essential preoperative assessment, prompting our selection of chest CT instead. Numerous studies (12–14) indicate that skeletal muscle assessments at the T12 level can facilitate the diagnose of sarcopenia. Additionally, alterations in muscle area at L3 during a 48-month period were often correlated with alterations at T12.

Sarcopenia was an independent prognostic factor in worse DFS (p = 0.001) and OS (p = 0.006), according to our findings. The association between sarcopenia and the prognosis of early-stage and non-metastatic breast cancer has been extensively researched in the past. Sarcopenia was identified by Xinyi Liu et al. (23) as an independent predictive factor for both OS (HR, 2.13; 95% CI, 1.00–4.51; p = 0.049) and DFS (HR,1.44; 95% CI, 1.02–2.03; p = 0.038) among 2,948 Chinese female patients with breast cancer. Analyzing 3,241 patients with non-metastatic breast cancer, Caan and colleagues (8) concluded that sarcopenia was also related to overall mortality (HR, 1.41; 95% CI, 1.18–1.69). As a result of studying the total skeletal muscle and fat tissue areas in two adjacent axial slices obtained at the L3 vertebra by CT in 119 female breast cancer patients, Elise Deluche et al. (22) discovered that sarcopenia is a risk factor for death among these women who were diagnosed with early breast cancer. On the other hand, Rier et al. (24) revealed that poor muscle radiodensity (HR, 1.72; 95%CI, 1.14-2.62) among patients with metastatic illness was significantly linked to an elevated risk of overall death, independent of sarcopenia. Therefore, we explored the impact of sarcopenia on the prognosis of patients with TNBC. By collecting 130 patients with TNBC, we discovered that the patients with sarcopenia had shorter DFS and OS compared to non-sarcopenia patients, indicating poorer prognosis and survival.

A number of theories have been put forth to explain the possible negative impact of sarcopenia on the prognosis of breast cancer. Loss of muscle mass and function is a hallmark of sarcopenia, which raises the risk of frailty, metabolic dysfunction, and comorbidities (20, 25). An imbalance in the metabolism of proteins results in a surge in apoptosis and a decrease in muscle cell renewal (26, 27). Numerous critical physiological functions, including insulin sensitivity, respiratory integrity, cardiac output, and glucose homeostasis, are influenced by muscle tissues (28). In skeletal muscle, insulin typically has a substantial anabolic effect by phosphorylating insulin receptor substrates through tyrosine kinase receptors and activating the PI3K/AKT pathway (29). Sarcopenia may worsen insulin resistance due to the loss of skeletal muscle, which could further raise the likelihood of negative outcomes for patients (30). Moreover, investigations have demonstrated that sarcopenia is highly associated to immunological and inflammatory pathways (31). Low muscle mass is substantially connected with a high neutrophil-to-lymphocyte ratio (NLR), signs of systemic inflammation, and poor survival, according to a cohort of patients with nonmetastatic colorectal cancer (32). Bian AL et al. (33) found that Sarcopenia is positively correlated with pro-inflammatory markers such as TNF-α, IL-6, fbrinogen, C-reactive protein, and fbrinogen, which may accelerate the development of breast cancer. Additionally, the existence of crown-like structures in the adipose tissue next to the tumor, a histological indication of local inflammation, is linked to tumor progression in breast cancer, providing more proof of the significance of the tumor microenvironment (34). Furthermore, sarcopenia is accelerated by age-related inflammation, creating a vicious cycle (35). What’s more, patients with sarcopenia experience a higher risk of severe chemotherapy toxicities, which may further raise the likelihood of poor prognosis and survival (36). In conclusion, the relationship between sarcopenia and the higher death rate among patients with breast cancer is nuanced, and further study is required to clarify the underlying mechanisms.

How can an effective intervention be carried out for TNBC patients with sarcopenia? Exercise is a non-invasive intervention that has been demonstrated to be the most effective means of maintaining optimal body composition. A substantial body of previous research has confirmed that exercise has a beneficial impact on body composition and also exerts a favorable influence on biological changes that may prevent cancer progression. Exercise raised blood levels of norepinephrine and adrenaline, which triggered the tumor-suppressive Hippo signaling pathway, according to a preclinical study (37). Exercise also has an anti-tumor effect because, according to this study, it phosphorylates and retains the oncoprotein YAP in the cytoplasm while also reducing the expression of downstream target genes. Furthermore, exercise intervention was found to enhance levels of metabolic biomarkers, such as adiponectin, leptin and insulin, as well as a variety of body composition assessments in randomized clinical trials (38–40).

The primary recommendation is for an aerobic exercise training (AET) and resistance exercise training (RET) intervention over a 16-week period, which has been demonstrated that they can improve systemic inflammation by reducing M1/M2 macrophage ratios and decreasing IL-6 and TNF-α secretion (41). With 9 workouts each set of eight to twelve repetitions, RET significantly reversed sarcopenia in a prospective randomized experiment with 200 breast cancer patients (42). Given that every patient with breast cancer has distinct conditions according to their age, physical health, body composition, and ongoing treatment, it is necessary to develop a thorough exercise plan tailored to individual needs to get the most benefit (43).

To maintain or improve body composition, nutritional support is just as important as exercise. Various solutions for dietary supplementation have been recommended to enhance body composition during this time. For example, it’s highly recommended to supplement with protein (44), branched-chain amino acids (45) and vitamin D (46). A well-designed clinical trial is necessary to obtain robust evidence on the impact of nutritional assistance on patients with breast cancer, as there is currently insufficient data supporting their significance in TNBC.

## Conclusion

Sarcopenia measurements from clinically obtained CT scans of TNBC patients offer important prognostic data that will direct therapies to optimize survival outcomes.

## Data Availability

The raw data supporting the conclusions of this article will be made available by the authors, without undue reservation.
